# Genetics and genomics of dilated cardiomyopathy and systolic heart failure

**DOI:** 10.1186/s13073-017-0410-8

**Published:** 2017-02-22

**Authors:** Upasana Tayal, Sanjay Prasad, Stuart A. Cook

**Affiliations:** 1 National Heart Lung Institute, Imperial College London, Cale Street, London, SW3 6LY UK; 2 Cardiovascular Biomedical Research Unit, Royal Brompton Hospital, Sydney Street, London, SW3 6NP UK; 3 Duke National University Hospital, 8 College Road, Singapore, 169857 Singapore

## Abstract

Heart failure is a major health burden, affecting 40 million people globally. One of the main causes of systolic heart failure is dilated cardiomyopathy (DCM), the leading global indication for heart transplantation. Our understanding of the genetic basis of both DCM and systolic heart failure has improved in recent years with the application of next-generation sequencing and genome-wide association studies (GWAS). This has enabled rapid sequencing at scale, leading to the discovery of many novel rare variants in DCM and of common variants in both systolic heart failure and DCM. Identifying rare and common genetic variants contributing to systolic heart failure has been challenging given its diverse and multiple etiologies. DCM, however, although rarer, is a reasonably specific and well-defined condition, leading to the identification of many rare genetic variants. Truncating variants in titin represent the single largest genetic cause of DCM. Here, we review the progress and challenges in the detection of rare and common variants in DCM and systolic heart failure, and the particular challenges in accurate and informed variant interpretation, and in understanding the effects of these variants. We also discuss how our increasing genetic knowledge is changing clinical management. Harnessing genetic data and translating it to improve risk stratification and the development of novel therapeutics represents a major challenge and unmet critical need for patients with heart failure and their families.

## Background

**Fig. 1:**
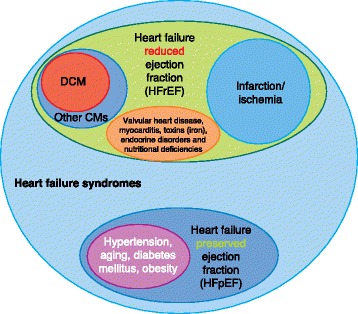
An overview of heart failure syndromes showing where dilated cardiomyopathy (DCM) and systolic heart failure fit in relation to all heart failure syndromes. Heart failure syndromes encompass clinical symptoms and/or signs of heart failure and evidence of myocardial dysfunction. This can occur in the setting of reduced (*HFrEF*; left ventricular ejection fraction <40%) or preserved (*HFpEF*; left ventricular ejection fraction >50%) left ventricular ejection fraction. The contribution of HFpEF, previously referred to as diastolic heart failure, to heart failure syndromes ranges from 22 to 73%, reflecting the difficulties in defining the condition and the diversity of the populations studied [8]. Recently, a third category of heart failure with mid-range ejection fraction (HFmrEF; left ventricular ejection fraction 40–49%) has been identified [8], although it has not yet been encompassed into clinical studies. The commonest cause of HFrEF is myocardial ischemia. DCM can be a subset of HFrEF and is the commonest cardiomyopathy (*CM*) to cause heart failure syndromes. Although DCM can present with the clinical syndrome of systolic heart failure, it can also present with arrhythmias or thrombo-embolic disease or be detected in the asymptomatic patient. DCM therefore does not equate with systolic heart failure. DCM is predominantly an imaging diagnosis, whereas heart failure is a clinical and imaging diagnosis. *DCM* dilated cardiomyopathy; *Other CMs* other cardiomyopathies, including hypertrophic cardiomyopathy

Heart failure is an umbrella term for a compendium of patient symptoms and physical-examination findings that are associated with impaired ventricular function, predominantly due to left ventricular systolic (contractile) dysfunction (Fig. 1; Box 1). Heart failure represents a final common phenotype in response to genetic and/or environmental insults and is thought to affect approximately 40 million people globally [1].

Conventionally categorized based on the level of ejection fraction as well as by the underlying cause (Fig. 1), heart failure is most commonly due to ventricular impairment following an ischemic insult, notably myocardial infarction followed by muscle necrosis, but is also seen with chronic myocardial hypo-perfusion.

The cardiomyopathies (intrinsic diseases of heart muscle), including dilated, hypertrophic and restrictive forms, can all lead to heart failure, although dilated cardiomyopathy (DCM) has particular importance as the leading global cause for heart transplantation [2–4]. DCM has an estimated prevalence of approximately 1:250, although this might be overestimated [5]. DCM can be a subset of systolic heart failure, and, although it can present with the clinical syndrome of systolic heart failure, it can also present with arrhythmias or thrombo-embolic disease or be detected in the asymptomatic patient. DCM therefore does not equate with systolic heart failure. DCM is predominantly an imaging diagnosis, whereas heart failure is a clinical and imaging diagnosis.

Heart failure due to hypertrophic cardiomyopathy (HCM) has been reviewed elsewhere [6] and is not discussed in detail here. Likewise, we do not discuss heart failure with preserved ejection fraction (HFpEF), which represents the situation whereby a patient has symptoms and signs of heart failure but ventricular systolic function is ostensibly normal [7]. Estimates of the contribution of HFpEF, previously referred to as diastolic heart failure, to heart failure syndromes range from approximately 20 to 70% of cases, reflecting the difficulties in defining the condition and the diversity of the populations studied [8]. Moreover, HFpEF is a highly heterogeneous disease, and genetic effects can be expected to be very limited as the disease is of late onset and associated with multiple environmental triggers, hence HFpEF is not discussed further.

Despite optimal medical therapy, clinical outcomes remain poor for patients with heart failure syndromes, with a 5-year mortality of 20% in DCM [9, 10]. Novel heart failure therapies beyond devices have recently emerged, but it is too soon to be able to evaluate their long-term prognostic benefit [11], and whether current therapies can be tailored to an individual patient has yet to be explored in detail [12]. Risk stratification tools in DCM are limited and largely based on qualitative clinical data, imaging features, and biochemical markers, many of which reflect changes observed late in the disease course. Faced with these difficulties, the ideal risk assessment tool would be one that identifies patients at risk of heart failure before overt disease at a time when a preventative intervention could be used to avoid disease onset. Genetics offers one such approach.

There have been major advances in DNA sequencing technologies over recent years, which have enabled the widespread application of DNA sequencing of heart failure cohorts. This has led to a rapid increase in the number of genes associated with DCM. At an even more rapid pace, DNA sequencing at scale has been applied in very large cohorts, such as those included in the Exome Aggregation Consortium (ExAC) data-set [13] [now renamed the Genome Aggregation Database (gnomAD) to reflect the inclusion of genome sequencing data]. Against this background, understanding which genes and variants are of importance for a patient with DCM, or indeed an apparently healthy individual, is a challenge for the clinician.

In this review, we examine the genetic underpinnings of heart failure syndromes, focusing on systolic heart failure and DCM. We summarize the advances in rare and common variant discovery and interpretation in DCM and systolic heart failure, placing recent discoveries in the context of early work. We reflect upon how these discoveries have changed patient management before considering what implications these findings hold for future research and patient care.

## The genetic architecture of heart failure syndromes is complex

The proportion of DCM cases with a familial basis is between 20 and 30%, although a level as high as 60% has been suggested [14]. In familial DCM, up to 40% of cases can have an identifiable genetic basis [5], although as a more critical evaluation of the genes linked to DCM continues and genes or variants are discounted, this percentage might fall [15, 16]. Systolic heart failure is a catch-all phenotypic diagnosis and can be caused by a variety of insults ranging from myocardial ischemia to cardiomyopathy. This lack of specificity limits our understanding of the contribution of genetic variants to systolic heart failure.

Rare variants are typically defined as having a minor allele frequency (MAF) of <1%, although the frequency cut-offs in the literature vary [17]. In line with current widely accepted definitions, we define rarity as an allele frequency of <0.001. However, for evaluation of potentially pathogenic variants, we recommend a disease-specific cut-off informed by disease prevalence, penetrance, and allelic contribution to disease [18, 19]. Rare variants are identified through next-generation DNA sequencing approaches such as targeted (panel-based) sequencing, whole-genome or whole-exome sequencing, or traditional Sanger capillary-based sequencing.

Common variants are typically defined as having a MAF of >5%. Common variants are identified by genotyping of single-nucleotide polymorphisms (SNPs) on sub-genome arrays (candidate gene studies) or chips containing many hundreds of thousands of SNPs that, together with imputation (a statistical process), provide genome-wide coverage. These approaches form the basis of genome-wide association studies (GWAS).

### Variable disease phenotyping

As with all genetic studies, careful phenotyping of the condition under investigation is crucial for accurate evaluation and to avoid confounding effects due to phenotypically similar, but etiologically distinct, conditions. Heart failure is particularly challenging as it encompasses heterogeneous conditions with diverse pathobiologies. DCM, although more limited in its definition, is not immune to imprecise phenotyping, depending on the imaging modality used [20], and has a heterogeneous underlying etiology as well as diverse forms at the imaging and genetic levels. Accurate phenotyping is therefore important to distinguish DCM from other causes of ventricular dysfunction. The study of heart failure as a whole does, however, permit the study of a ‘final common pathway’ of myocardial damage common to cardiomyopathies, ischemia, and toxic insults.

### Challenges in the interpretation of genetic variants

The interpretation of potentially disease-causing rare variants is challenging owing to the relatively high frequency of rare benign variation in the population. This means that an individual variant might be rare (allele frequency <0.001) but, collectively, variation in a specific gene is common. For example, healthy individuals appear to carry many unique (private) variants that do not cause disease. There is, therefore, a need for robust population-matched control data to avoid spurious gene–disease associations. The ExAC data-set of over 60,000 exomes will help to address the pressing need for greater amounts of control data [13]. Several groups have shown how ExAC can be leveraged to aid the interpretation of rare variants in cardiomyopathies [15, 16]. These population data should be placed, however, in the context of other available resources to aid clinicians and researchers in interpreting rare variants, such as disease variant databases (for example, Human Gene Mutation Database [21] and ClinVar [22]), computational data (such as in silico missense variant prediction tools, many of which are amalgamated in the dbNSFP [23]), functional data, and, crucially, segregation data. Conflict can arise between these sources, leading to a greater proportion of variants being categorized as of ‘uncertain significance’ instead of ‘likely pathogenic’ or ‘pathogenic’. We direct the reader to the recent American College of Medical Genetics and Genomics report that provides comprehensive guidelines on variant interpretation [24].

## Genetic variants affecting systolic heart failure

In this section, we review advances in the genetics of systolic heart failure, beginning with a brief discussion of why discovery of rare variants in systolic heart failure has been limited, then moving on to a brief summary of candidate gene studies that underpinned the early discovery work in this field, before focusing on the advances yielded from the study of common variants in systolic heart failure using GWAS.

### Rare variants

Heart failure has a heritable component, estimated at 18% based on analyses of the Framingham data-set [25]. However, excluding the monogenic cardiomyopathies that are due to very rare, private or novel alleles, the contribution of rare variants (allele frequencies <0.001) to the risk of systolic heart failure is likely limited and has yet to be shown conclusively. This is because, as highlighted above, the etiology of systolic heart failure is complex and each associated condition might have its own genetic basis (for example, hypertension and diabetes), making it hard to distinguish primary from secondary effects [26]. Genes that are linked to primary cardiomyopathies might play little or no role in common heart failure, but could serve to highlight molecular pathways that are important for heart failure syndromes more generically [27].

### Candidate gene studies

Many of the published genetic studies of heart failure have been candidate gene studies for genes involved in the adrenergic and renin-angiotensin-aldosterone pathways that are important for heart failure pathobiology. However, the most promising associations suggested by the early candidate gene studies are now no longer thought to be informative. For example, a meta-analysis of 17 case-control studies assessing the angiotensin-converting enzyme insertion/deletion polymorphism (ACE I/D) found no association with heart failure [28]. Similarly, a meta-analysis of 27 studies evaluating the link between common beta 1 adrenergic receptor polymorphisms (Ser49Gly and Arg389Gly) and heart failure, first reported in 2000 [29] and 2003 [30], found that neither was an independent predictor of prognosis in heart failure [31]. Candidate gene methodologies have now largely been replaced by the unbiased approach of GWAS.

### Common variants

The study of common variants in systolic heart failure has had some success. Table 1 highlights two studies of common variants associated with heart failure that are specific to the heart failure phenotype. Here, we discuss GWAS approaches to identify variants associated with potential biomarkers and phenotypes associated with heart failure, and examine how further studies of the identified variants can provide insights.

**Table 1:** Summary of genome-wide association studies for heart failure and dilated cardiomyopathy

Study	Study design	Disease^a^	Discovery cohort	SNP	SNP location	Replication cohort	Nearest gene
CHARGE Consortium [32]	Meta-analysis Case control	Incident systolic heart failure	20,926 European-ancestry individuals and 2895 African-ancestry individuals followed up for incident heart failure events	rs10519210 (European) rs11172782 (African)	Intergenic Intergenic	–	*USP3* (European) *LRIG3* (African)
Cappola et al. [38]	Case control; 2000 genes pre-selected for cardiovascular relevance	Advanced heart failure	1590 Caucasian patients with heart failure 577 controls	rs1739843 rs6787362	Intronic Intronic	308 cases 2314 controls	*HSPB7* *FRMD4B*
Villard et al. [39]	Case control	DCM	1179 DCM patients 1108 controls	rs10927875 rs2234962	Intronic Coding	1165 DCM patients 1302 controls	*ZBTB17* *BAG 3*
Meder et al. [73]	Case control	DCM	909 DCM patients 2120 controls	rs9262636	Intronic	Within study, between cohorts First replication - in 2597 DCM cases, 4867 controls Second replication; lead SNP was replicated in a cohort of 637 DCM cases and 723 healthy controls	*HCG22* eQTL for class I and class II MHC receptors
Stark et al. [41]	Case control; 2000 genes pre-selected for cardiovascular relevance	Idiopathic DCM	664 DCM cases 1874 controls	rs1739843	Intronic	Genotyping of lead SNPs in three independent case-control studies of idiopathic DCM Cases 564/433/249 Controls 981/395/380	*HSPB7*

^a^For heart failure, the table focuses on the two main heart failure-specific studies with the strongest evidence. Refer to the main text for discussion of studies evaluating cardiac endophenotypes, quantitative proxy markers, or subgenome array studies

One of the first GWAS of heart failure was carried out by the CHARGE (Cohorts for Heart and Aging Research in Genomic Epidemiology) consortium [32]. In this meta-analysis of four large community-based cohort studies, almost 25,000 individuals were followed up for a mean of 11.5 years for the development of incident (new onset) heart failure. This study identified two loci, one that was near to the gene *USP3* (encoding ubiquitin-specific peptidase 3) in individuals of European ancestry, and one near to the gene *LRIG3* (encoding leucine-rich repeats and immunoglobulin-like domains 3) in individuals of African ancestry. These findings have yet to be replicated and as such their importance has yet to be clarified.

Evaluations of a quantitative marker of heart failure severity or an endophenotype associated with heart failure, both described below, are alternative approaches to the study of systolic heart failure, and might mitigate some of the limitations of imprecise phenotyping of ‘heart failure’ per se.

Cardiac hypertrophy is a common end-result of heart failure but is a very complex phenotype. One GWAS identified a SNP associated with cardiac hypertrophy (rs2207418, *P* = 8 × 10^–6^) that was then studied in a heart failure case-control cohort and was found to associate with both heart failure and heart failure mortality [33]. This SNP is located in a gene desert on chromosome 20, although near a highly conserved region. The implications are that this region might be biologically important, but the mechanism of action is yet to be established.

Levels of N-terminal pro-brain natriuretic peptide (NT-proBNP) increase with myocardial wall stress and are associated with heart failure. A quantitative GWAS of NT-proBNP levels was performed, although this was measured in the general population and not a heart failure population [34], and it is worth noting that NT-proBNP levels might equally be regulated by genetic factors unrelated to heart failure. From a discovery cohort of 1325 individuals and a replication cohort of 1746 individuals, the *CLCN6* gene was independently associated with NT-proBNP levels (rs 1023252, *P* = 3.7 × 10^–8^). *CLCN6* encodes a voltage-gated chloride channel. Indeed, *CLCN6* might not be mechanistically implicated in heart failure at all but instead it might modify expression of *NPPB* (the gene encoding BNP) in *trans*, or might directly regulate *NPPB* in *cis* given the strong linkage disequilibrium (LD) at the locus. It is yet to be established whether the results of this GWAS, identifying the *CLCN6* gene and its possible interaction with *NPPB*, have clear mechanistic implications for the study of the pathogenesis of systolic heart failure.

Other GWAS have evaluated the association between common variants and cardiovascular endophenotypes of left ventricular dimensions, function, and mass assessed by echocardiography or cardiac magnetic resonance imaging (MRI). The largest of these focussed on an African-American population of 6765 individuals derived from four community-based cohorts [35]. The study identified four genetic loci at genome-wide significance (4.0 × 10^−7^) that were associated with cardiac structure and function. SNP rs4552931 (*P* = 1.43 × 10^−7^) was associated with left ventricular mass. The nearest gene is *UBE2V2* (which encodes ubiquitin-conjugating enzyme E2 variant 2), involved in protein degradation. An intronic SNP on chromosome 10 was associated with interventricular septal wall thickness (rs1571099, *P* = 2.57 × 10^−8^), and an intergenic SNP on chromosome 17 was associated with left ventricular internal diastolic diameter (rs7213314, *P* = 1.68 × 10^−7^). Finally, rs9530176, near the *CHGB* gene (encoding chromogranin B), was associated with left ventricular ejection fraction (*P* = 4.02 × 10^−7^). This protein is abundant in human catecholamine secretory vesicles and might play a role in modulation of catecholamine secretion. However, these variants did not replicate in the EchoGEN European cohort that the authors also investigated [35].

A recent, novel approach to evaluating genetic determinants of myocardial hypertrophy has been to evaluate electrocardiographic (ECG) proxy markers of hypertrophy [36]. The advantages of this are that, compared with imaging (using echocardiography or cardiac MRI), ECG is rapidly acquired, systematically quantifiable, and low cost. In this meta-analysis of over 73,000 individuals, 52 genomic loci were identified as being associated with ECG markers of hypertrophy (QRS traits; *P* < 1 × 10^–8^). Although a comprehensive evaluation of these loci is beyond the scope of this review, it is interesting that, of these loci, 32 were novel, and in total 67 candidate genes were identified that were expressed in cardiac tissue and associated with cardiac abnormalities in model systems. These loci appeared to play a role in cardiac hypertrophy. Further study of these loci is required to locate the causal genes and molecular pathways leading to the development of cardiac hypertrophy.

One shortcoming of the GWAS approach is that real genetic associations might not pass stringent genome-wide corrected significance thresholds. Using a candidate gene approach to investigate variants that might not pass this threshold in GWA studies is one way to mitigate multiple testing effects. For example, a study evaluating 77 SNPs in 30 candidate genes, most linked to inflammation, evaluated a mixed Caucasian heart failure population (322 DCM patients, 268 ischemic cardiomyopathy patients) and found a 600-kb region on chromosome 5 to be associated with cardiomyopathy (combined *P* = 0.00087) that replicated in two further populations [37]. The authors performed zebrafish studies that revealed the disruption of three genes (*HBEGF*, *IK*, and *SRA1*) in this region that led to a phenotype of myocardial contractile dysfunction. The authors sought to challenge the paradigm that association studies identify a single causal or susceptibility locus, and instead point to a haplotype block that is associated with heart failure. A similar, but expanded, candidate gene study used subgenome analysis of approximately 50,000 SNPs in approximately 2000 genes linked to cardiovascular disorders. In this study, two SNPs were associated with advanced heart failure in the discovery and replication cohorts [38] (Table 1). Of these, the most significantly associated SNP for both ischemic and non-ischemic heart failure was located in an intronic region of the *HSPB7* gene.

HSPB7 warrants some further discussion as it has been identified in studies of both heart failure and DCM [39, 40]. HSPB7 is a member of the small heat-shock protein family, expressed in cardiac and skeletal muscle, and functions to stabilize sarcomeric proteins (Box 1). This same locus was also identified in a GWAS of DCM [41], which could reflect either the physiological importance of *HSPB7* and/or the likelihood that DCM patients were a subset of the heart failure patients. It is important to note, however, that the original SNP (rs1739843) and subsequent SNPs in *HSPB7* that were associated with heart failure were intronic or synonymous. The *CLCNKA* gene, encoding the renal ClC-Ka chloride channel, is in high LD with *HSPB7*. A common SNP (rs10927887) in *CLCNKA* is associated with both ischemic and non-ischemic heart failure and increased risk of heart failure (odds ratio 1.27 per allele copy) [42]. In an expression quantitative trait locus (eQTL) study of DCM, *HSPB7* SNPs were associated with expression of both the *HSPB7* and the *CLCNKA* gene (rs945425, *HSPB7* expression *P* = 6.1 × 10^–57^, *CLCNKA* expression *P* = 2.2 × 10^–26^) [39]. Therefore, the identification of *HSPB7* could reflect the potentially important role of the heat-shock protein itself (HSPB7), or the importance of the renal ClC-Ka chloride channel. The latter is particularly interesting as it alludes to a multisystem biology of heart failure pathogenesis, something that is clinically well established.

In summary, a number of studies have been performed to identify and evaluate causal or susceptibility variants in heart failure syndromes, but as yet no consistent themes or common pathways are emerging. Susceptibility variants are located in both cardiac genes (for example, *HSPB7*) and non-cardiac genes (for example, the renal chloride channel *CLCNKA*). Modulators of catecholamine secretion, cell signaling, and protein degradation have all been implicated, suggesting complexity of the underlying mechanism(s). Studies to date have also demonstrated the limitation of the variable phenotyping that is associated with the ‘heart failure’ syndrome. There has been increasing success in studying cardiovascular endophenotypes of the heart failure syndrome, such as myocardial mass or biomarker levels, and this might be the most promising avenue for future advances.

## Genetic factors affecting dilated cardiomyopathy

Here, we review advances in our understanding of the contribution of rare and common variants to DCM. We focus particularly on rare variants, given the growth in the number of variant genes implicated in DCM, and the challenges in interpreting these data. There have been fewer advances from common variant studies of DCM, and we summarize briefly two of the major DCM GWAS.

### Rare variants

Rare genetic variants associated with DCM have been identified in genes involved with a range of diverse cellular structures and functions, and most notably with the sarcomere (Table 2). Inheritance of DCM is most commonly autosomal dominant, although autosomal recessive, X-linked, and mitochondrial inheritance have also been reported, particularly in pediatric populations [43]. Approximately 40% of familial DCM is thought to have a primary monogenic basis [5]. Higher estimates of sensitivity for genetic testing have been reported (from 46 to 73% in one study [44]), but these estimates are likely confounded by insufficient control for population variation in the genes studied. Although variants in over 50 genes have been linked to DCM, the evidence is most robust for a ‘core disease set’ encompassing the sarcomeric genes *MYH7* (which encodes beta myosin heavy chain), *TNNT2* (which encodes troponin T2), and *TTN* (encoding titin) and the gene *LMNA* encoding a nuclear envelope protein.

**Table 2:** Genes implicated in monogenic dilated cardiomyopathy and their cellular component

Gene	Protein	Function	Estimated contribution in DCM patients and phenotypic comments
Sarcomeric			
MYH7*	Myosin-7 (beta myosin heavy chain)	Muscle contraction	Non-truncating variants: 5%
TNNT2*	Troponin T, cardiac muscle (troponin T2)	Muscle contraction	Non-truncating variants: 3%
TTN*^,#^	Titin	Extensible scaffold/molecular spring	Truncating variants: 15–25%
TPM1*	Tropomyosin alpha-1 chain	Muscle contraction	<2%
*MYBPC3*	Myosin-binding protein C, cardiac type	Muscle contraction	Major hypertrophic cardiomyopathy gene; purported association with DCM now less likely in light of population variation data [16]
*TNNC1*	Troponin C, slow skeletal and cardiac muscles	Muscle contraction	Mutations also associated with hypertrophic cardiomyopathy
*TNNI3*	Troponin I, cardiac muscle	Muscle contraction	Mutations also associated with hypertrophic cardiomyopathy
*MYL2* ^#^	Myosin regulatory light chain 2, ventricular/cardiac muscle isoform	Regulation of myosin ATPase activity	Mutations also associated with hypertrophic cardiomyopathy
*FHOD3* ^#^	FH1/FH2 domain-containing protein 3	Sarcomere organization	
Cytoskeleton			
DES*	Desmin	Contractile force transduction	<1%
DMD*	Dystrophin	Contractile force transduction	In patients with dystrophinopathies. X-linked
*VCL*	Vinculin	Cell–matrix and cell–cell adhesion	
Nuclear envelope			
LMNA*	Prelamin-A/C	Nuclear membrane structure	4%
Mitochondrial			
*WWTR1 (TAZ)*	Tafazzin (WW domain-containing transcription regulator protein 1)		Associated with syndromic DCM (for example, Barth syndrome). X-linked
Spliceosomal			
*RBM20*	RNA-binding protein 20	Regulates splicing of cardiac genes	2%
Sarcoplasmic reticulum			
*PLN*	Cardiac phospholamban	Sarcoplasmic reticulum calcium regulator; inhibits SERCA2a pump	<1% Linked to an arrhythmogenic phenotype
Desomosomal			
DSP*	Desmoplakin	Desmosomal junction protein	Truncating variants: 3% Linked to arrhythmogenic right and left ventricular cardiomyopathy
*DSC-2* ^#^	Desmocollin-2	Desmosomal junction protein	Linked to arrhythmogenic right and left ventricular cardiomyopathy
*DSG2* ^#^	Desmoglein-2	Desmosomal junction protein	Linked to arrhythmogenic right and left ventricular cardiomyopathy
*PKP2* ^#^	Plakophilin-2	Desmosomal junction protein	Linked to arrhythmogenic right and left ventricular cardiomyopathy; recent studies cast doubt on involvement in DCM
*JUP*	Junction plakoglobin	Desmosomal junction protein	Linked to arrhythmogenic right and left ventricular cardiomyopathy
Ion channels			
*SCN5A*	Sodium channel protein type 5 subunit alpha	Sodium channel	<2%. Associated with atrial arrhythmias and conduction disease. Association with DCM in absence of segregation less strong in light of population variation data [16]
Z-disc			
*FLNC* ^#^	Filamin-C	Structural integrity of cardiac myocyte; actin crosslinking protein	–
*NEBL*	Nebulette	Z-disc protein	–
*NEXN*	Nexilin	Encodes a filamentous actin binding protein	–
*CSRP3*	Cysteine and glycine-rich protein 3	Mechanical stretch sensing	–
*TCAP*	Telethonin	Mechanical stretch sensing	–
*LDB3*	Lim domain-binding 3	Z-disc structural integrity	Associated with left ventricular non-compaction phenotypes
*CRYAB*	Alpha-crystallin B chain	Heat-shock protein	
Other			
*BAG3* ^#^	BAG family molecular chaperone regulator 3	Inhibits apoptosis	–
*ANKRD1*	Ankyrin repeat domain-containing protein 1	Encodes CARP, a transcription coinhibitor	<2%
*RAF1* ^#^	RAF proto-oncogene serine/threonine-protein kinase	MAP3 kinase, part of the Ras–MAPK signaling cascade	~9% in childhood-onset DCM (one study)
Transcription factors			
*PRDM16* ^#^	PR domain zinc finger protein 16	Transcription factor	Mutations cause cardiomyopathy in 1p36 deletion syndrome; also linked to isolated DCM and left ventricular non-compaction
*ZBTB17* ^#^	Zinc-finger and BTB domain-containing protein 17	Transcription factor	
*TBX5* ^#^	T-box transcription factor TBX5	Transcription factor	Associated with congenital heart disease; also linked to adult-onset DCM
*NKX2-5* ^#^	Homeobox protein Nkx-2.5	Transcription factor	Associated with congenital heart disease; also linked to adult-onset DCM
*GATA4* ^#^	Transcription factor GATA-4 (GATA-binding protein 4)	Transcription factor	Linked to sporadic and familial DCM
*TBX20* ^#^	T-box transcription factor TBX20	Transcription factor	Associated with congenital heart disease; also linked to adult-onset DCM

Table content adapted from Hershberger et al. [5] and Walsh et al. [16]. We have highlighted the genes with the strongest evidence linking them to dilated cardiomyopathy (DCM; marked with an asterisk) or the most recently identified genes from 2011 onwards (marked with a hash sign). Causes of predominantly autosomal recessive DCM and older gene associations that have not been replicated have not been included

A recent large-scale analysis of rare genetic variation in cardiomyopathy cases compared with normal population variation has also provided insights into the genetics of DCM. The study tested for an excess of rare variants in 46 genes sequenced in up to 1315 DCM cases compared with over 60,000 ExAC reference samples. Truncating variants in *TTN* were the most common DCM rare variant (14.6%) [16]. There was modest, statistically significant enrichment in only six other genes (*MYH7*, *LMNA*, *TNNT2*, *TPM1*, *DSP*, and *TCAP*) (Table 2). Based on available data, *RBM20* is also likely to prove significant (reviewed below) but was not included in the published analysis owing to poor coverage in the ExAC data. Furthermore, sequencing methods were not uniform, and not all genes were sequenced across the DCM cohorts included in the study. Even allowing for this, many genes that have previously been linked to DCM, including genes routinely sequenced in clinical practice such as *MYBPC3* and *MYH6*, showed little or no excess burden in DCM compared with the reference population. The accompanying Atlas of Cardiac Genetic Variation web resource [16] summarizes these data and serves as a useful adjunct to facilitate the interpretation of rare variants in DCM.

#### Recent disease–gene associations in DCM

Over the past decade, 47 new genes have been categorized as linked with DCM in the Human Gene Mutation Database (HGMD). Many of these links have not been replicated outside of the original reports, and a comprehensive review of these is beyond the scope of this article. A few examples of novel associations are discussed below, selected for critical evaluation either owing to robust evidence, novelty, or clinical importance.

*BAG3* encodes a heat-shock chaperone protein and was first linked to DCM in 2011 through the discovery of a large 8733-bp deletion in exon 4 in seven affected family members in a three-generation family, which was absent in 355 controls [45]. Subsequently, coding exons in *BAG3* in 311 other unrelated DCM probands were sequenced, which identified seven rare variants (one frameshift, two nonsense, and four missense variants) that were absent from 355 controls. The authors were also able to recapitulate the DCM phenotype in a zebrafish *bag3* knockdown model. In separate studies, *BAG3* was linked to DCM through a GWAS, with the discovery of a non-synonymous SNP in the coding sequence of *BAG3* in DCM cases compared with healthy controls, which is discussed further below (rs2234962, *P* = 1.1 × 10^–13^) [39]. The authors then performed targeted sequencing in a cohort of 168 unrelated DCM probands and identified six variants that were also detected in affected relatives, lending further support to the role of *BAG3* as a disease-causing gene.

*RBM20* encodes a spliceosome protein that regulates pre-mRNA splicing for many genes, including *TTN* [46], which is why variants in this gene could hold particular relevance for DCM, either in isolation or in compound heterozygosity with *TTN* [47]. *RBM20* was initially associated with DCM through linkage analysis in two large families with DCM [48]. The authors sequenced all 14 *RBM20* exons in each family member and identified a heterozygous missense mutation in exon 9 that co-segregated with disease in all affected individuals, and that was absent in unaffected relations and 480 ethnically matched controls. The authors went on to detect *RBM20* missense mutations in exon 9 in six more families affected with DCM. Since the original link with DCM [48], subsequent studies found mutations both within and outside the original *RBM20* hotspot in DCM probands, but the segregation data on these variants is limited and the control population was modest in size, meaning that population-level missense variation was not accounted for in these regions [49, 50]. The association of *RBM20* and DCM appears most robust for variants in the original hotspot, and further curation is needed to understand the significance of variants in other regions.

The 1p36 deletion syndrome can be associated with cardiomyopathy, and the *PRDM16* gene (which encodes a transcription factor) has been identified as a possible cardiomyopathy gene at this locus, linked with a syndromic cardiomyopathy as well as with adult-onset DCM (in 5 out of 131 individuals with four novel missense variants) [51]. However, although there might be a role for *PRDM16* in cardiac development, its role as a cardiomyopathy gene has subsequently been questioned [52].

*ZBTB17* is also encoded on chromosome 1, at the 1p36 locus. A study of cardiac myocytes and a mouse model of *ZBTB17* deletion demonstrated that *ZBTB17* is involved in cardiac myocyte hypertrophy and is essential for cell survival [53]. The authors also showed that *ZBTB17* encodes a transcription factor (zinc-finger and BTB domain-containing protein 17) that binds the gene *CSRP3*, a Z-disc protein, mutations of which are found in both HCM and DCM. Given the association between *CSRP3* and DCM (in a small cohort with limited segregation data [54], with no subsequent replication), and this new-found function of *ZBTB17* in binding CSRP3, the authors hypothesized that *ZBTB17* could be a novel gene implicated in DCM.

Many additional transcription factors have also been linked to DCM in recent years, such as *GATA5* [55], *TBX20* [56], *TBX5* [57], *GATA6* [58], *GATA4* [59], and *NKX2*-5 [60]. Some of these genes are clearly linked to congenital heart disease phenotypes. However, many of the variants with claimed associations with DCM are missense variants that have been identified within one relatively small group of DCM patients, with variable segregation data. Further studies are required to confirm the link with DCM.

Desmosomal proteins, typically perturbed in arrhythmogenic right ventricular dysplasia/cardiomyopathy (ARVD/ARVC), have also been linked to DCM. The association has been most robust for *DSP*, which encodes desmoplakin, a desmosomal protein [61], with a strong excess of truncating variants in *DSP* in DCM [16]. However, some of the more recent associations of desmosomal protein gene variants have limited variant curation and segregation data, such as *PKP2* [62] (which encodes plakophilin 2), and these associations are less clear. One such *PKP2* variant (c.419C > T(p.(S140F)), previously linked to DCM has been shown not to be associated with heart failure phenotypes [63]. Therefore, of the desmosomal proteins, *DSP* variants have the most robust association with DCM.

Filamin-C (encoded by *FLNC*) is a Z-disc protein (Box 1) that provides sarcomeric stability. In recent work, two rare splicing variants in *FLNC* were detected through whole-exome sequencing in two Italian families and in one US family affected with DCM, with all variants co-segregating with disease [64]. Only one unaffected variant carrier was identified, but this individual declined further follow-up. These variants were absent from 1000 Genomes, NHLBI Go-ESP, and ExAC. The *FLNC* cardiomyopathy phenotype was not associated with skeletal muscle involvement in this cohort, but was associated with arrhythmias and sudden cardiac death. In the same study, a zebrafish knockdown model showed a phenotype of cardiac dysfunction, with defects in the Z-discs and sarcomere disorganization. Evaluation of *FLNC* variants in a large (*n* = 2877) cohort of patients with inherited cardiac diseases, including DCM, has shown that the phenotype of individuals with truncating variants in *FLNC* is notable for left ventricular dilation, systolic impairment, ventricular arrhythmias, cardiac fibrosis, and sudden cardiac death [65]. Further replication in DCM-specific cohorts is needed to validate this potentially prognostically important phenotypic association.

In summary, there have been many novel gene and variant associations with DCM. Although some appear robust and potentially clinically important (such as *FLNC*, *BAG3*, *RBM20*), others require further study (for example, variants in transcription factors). We encourage the reader to maintain critical review of variants outside of major disease genes and to utilize the variant interpretation aids we highlight in this article.

#### Truncating variants in titin

Truncating variants in the titin gene (*TTN*) represent the largest genetic cause of DCM, and, unlike many of the other genes related to DCM, a cardiologist is likely to encounter a DCM patient with one of these variants. However, as the interpretation of these variants is nuanced, we take the opportunity to discuss these variants in more detail. Variants in titin were first associated with DCM in 2002 through the study of two large multigenerational families affected with DCM [66]. In the first kindred, linkage analysis identified a disease gene locus [maximum logarithm of odds (LOD) score 5.0, penetrance of 70%]. In this study, *TTN* was chosen as a candidate gene owing to high levels of cardiac expression and its established role in muscle assembly and function. A 2-bp insertion was identified in exon 326 that resulted in a frameshift mutation generating a premature stop codon, and this mutation segregated with disease in family members. In the second kindred, a non-truncating *TTN* missense mutation in a highly conserved region was identified that also segregated with disease (Trp930Arg).

More recently, next-generation sequencing technologies have made the study of the giant titin gene (comprising 363 exons) possible in large cohorts. This led to the discovery that truncating variants in TTN (TTNtv) are found in approximately 15% of unselected DCM cases and in up to 25% of end-stage DCM cases [67, 68]. As yet, there do not appear to be any clear genotype–phenotype correlations permitting the phenotypic differentiation of genetic DCM, although one recent study suggests a milder phenotype associated with *TTNtv* cardiomyopathy than with non*-TTNtv* cardiomyopathy [69]. However, the findings in this latter study were driven by a direct comparison with *LMNA* cardiomyopathy, which has a severe and malignant phenotype, and need to be interpreted with this in mind.

Variant interpretation is complicated by the fact that *TTN* undergoes extensive alternative splicing to produce different protein isoforms, meaning that not all exons are included in the final processed mRNA transcripts. Allowing for this process, which is quantified by assessing the percentage spliced in (PSI)—that is, the percentage of final cardiac transcripts that include a particular exon—appears to be important for distinguishing variants that are important for disease. Variants in exons that are included in the final transcript more than 90% of the time are most significant for human cardiomyopathy [68]. Insights from induced pluripotent stem cell (iPSC) work suggest that the mechanism underlying TTNtv DCM might involve haploinsufficiency [70] as opposed to a dominant-negative model. The importance of haploinsufficiency was highlighted further in two rat models of TTNtv and by using Ribo-seq (integrated RNA sequencing and ribosome profiling) analysis of human RNA samples, which demonstrated haploinsufficiency of the mutant allele [71].

The finding of the importance of compound-heterozygous variants for severe phenotypes (for example, *TTN* and *LMNA* variants [72]) shows a potential for modifier genes or additive genetic effects in DCM. This concept was alluded to in a multi-center study of 639 patients with sporadic or familial DCM, with the finding of a 38% rate of compound mutations, and up to 44% when considering patients with TTNtv [44]. However, these findings must be interpreted with great caution as the ‘yield’ of DCM variants in this study was far higher than in any previous study, background population variation was not well accounted for, and there were no matched controls on the same sequencing platform.

### Common variants

There have been two notable DCM-specific case-control GWA studies, and their results are summarized in Table 1 [39, 73]. In the first of these studies, two SNPs with significant association to disease were discovered and replicated [39]. One SNP was located within the coding sequence of *BAG3* (rs2234962, *P* = 1.1 × 10^–13^), and the authors went on to identify rare variants in *BAG3* in a separate cohort of patients with DCM, as previously outlined. This is an unusual example of a situation where common and rare variants in the same gene can be associated with sporadic and monogenic forms of the disease, respectively. The second SNP was located within an intron of transcription factor gene *ZBTB17* (rs10927875, 3.6 × 10^–7^) [32]. *ZBTB17* has since been postulated to be involved in cardiomyopathy in a mouse model, as discussed above [53]. However, the genomic region of this second locus contains many other genes, including heat-shock protein gene *HSPB7*, which has been linked to heart failure syndromes multiple times.

In the second GWAS of DCM, SNPs in the *HSPB7* locus had weak association signals (rs1763610, *P* = 0.002; and rs4661346, *P* = 0.024) [73], but, in a separate association study of a subset of patients who featured in the replication stage of this GWAS, a stronger association was detected (rs1739843, *P* = 1.06 × 10^–6^) [41]. Taking these findings together with the findings of the sub-genome array studies of heart failure discussed above [38], a role for *HSPB7* in both DCM and heart failure is suggested. Also, in the second of the GWA studies for DCM, the most significant associated SNP (rs9262636, *P* = 4.9 × 10^–9^) was an eQTL for genes encoding class I and class II major histocompatibility complex heavy chain receptors [73]. This suggests that DCM might arise in part as a result of a genetically driven inflammatory process.

In summary, these GWAS in DCM identify susceptibility variants in genes with broad cellular functions (heat-shock proteins and inflammatory pathway receptors). This breadth makes interpretation of these findings challenging. Below, we discuss the potential translational implications of these data, and of the other rare and common variant discoveries in DCM and systolic heart failure.

## Translational implications

### Heart failure

As discussed above, many recent genetic studies of systolic heart failure have suggested the involvement of novel genes and loci. Although no clear new mechanistic pathways or novel drug targets have emerged from these studies, one of the most striking findings has been that, among those genes linked to systolic heart failure, not all are expressed exclusively within the heart. For example, the *CLCKNA* gene encodes a chloride channel in the kidney. The cardio–renal axis is well established clinically, but the identification of a possible genetic basis in heart failure offers cautious optimism that further study might reveal new therapeutic targets.

### Dilated cardiomyopathy

With regards to the potential development of novel and/or stratified therapeutic interventions, the HCM research field has led with the development of small-molecule inhibitors to suppress the development of genetic HCM in mice [74]. In this work, a small molecule (MYK-461) is able to reduce myocyte contractility, and, when administered to mice with HCM-causing myosin heavy chain mutations, suppresses the development of ventricular hypertrophy, myocyte disarray, and fibrosis, the hallmark features of HCM. This could mark the beginning of stratified medicine in HCM with treatment based on sarcomere mutation status.

Recent genetic advances in DCM have increased our understanding of DCM by providing new insights into the molecular mechanisms for disease pathogenesis. However, one of the key challenges in interpreting this mass of data will be to understand which genes are ‘causal’ drivers that directly lead to DCM, and which genes are less directly impactful and function more as susceptibility genes. Conceptually, it might be possible to correct the latter, restoring cardiac function.

In terms of correcting the ‘causal’ driver, one key example is the study of the *DMD* gene, encoding dystrophin, which is associated with X-linked DCM (Table 2) [14]. Like *TTN*, it is a large gene. The work by Olson and colleagues in animal models of gene editing to restore dystrophin expression in muscular dystrophy offers an insight into what might one day be achieved in DCM [75].

Next-generation sequencing methods have improved the efficiency and reduced the cost for genetic testing of diseases, including cardiomyopathies [76]. The increasing understanding of the genetic basis of DCM has highlighted the importance of considering genetic testing in all patients with DCM, not just those with a family history or a particular phenotype.

Although genetic testing can be carried out using multi-gene panels, in the clinical as opposed to research environment, we believe that analysis should be restricted to the genes known to be associated with DCM. One recent study showed that strict variant classification can facilitate a highly accurate diagnostic yield in DCM, with a pathogenic/likely pathogenic variant detection rate of 35.2% (47.6% in familial DCM and 25.6% in sporadic DCM) [61]. Even with these restrictions, many variants of uncertain significance (VUSs) are identified, particularly in genes with weak evidence linking them to DCM. In one study of a diagnostic sequencing laboratory, increasing the DCM gene panel from 5 to 46 genes increased the clinical sensitivity from 10 to 37%, but at the cost of a large increase in the number of VUSs, with the number of inconclusive cases rising from 4.6 to 51% [77]. By taking into account the amount of cumulative population-level rare variation in cardiomyopathy genes, the Atlas of Cardiac Genetic Variation website [16] is a resource to inform clinicians about the role of a specific gene in DCM or the status of an individual variant, aiding the assessment of the likelihood of pathogenicity.

Titin poses great challenges, as curation of variant pathogenicity depends upon additional information, such as whether an exon is constitutively expressed [68]. The fact that approximately 1% of apparently healthy individuals carry potentially pathogenic truncating variants in *TTN* highlights that we should currently only be interpreting these variants in individuals already known to have disease. An online resource has been developed to facilitate interpretation of TTN truncating variants in DCM patients [16, 68, 78]. This details the exon composition of the major TTN transcripts, with details of the PSI and other structural features for each exon, as well as the distribution of TTN variants in large published studies of cohorts of DCM patients and controls.

The discovery that peripartum cardiomyopathy shares a genetic etiology with DCM suggests that pregnancy might act as an environmental modifier to unmask the phenotype of TTNtv cardiomyopathy [79]. It has also been demonstrated that truncating variants of TTN are penetrant in apparently healthy humans, with subtle expressive changes in cardiac volumes compared with those of control subjects without TTNtv [71]. Furthermore, it was shown that rats with TTNtv developed impaired cardiac physiology under cardiac stress [71], providing further evidence of the importance of gene–environment interactions in the development of the TTNtv cardiomyopathy.

According to current expert recommendations, the primary role of the identification of a disease-associated genetic variant in patients with DCM (and indeed the other genetic cardiomyopathies) is to facilitate cascade screening and the early discharge of relatives who do not carry the variant in question [80]. For patients with DCM, conduction disease, and identified *LMNA* variants, clinical guidance suggests that an implantable cardiac defibrillator should be considered in preference to a conventional pacemaker owing to the identified genotype–phenotype correlation of an increased risk of malignant (potentially life-threatening) arrhythmias and sudden cardiac death [81].

The expansion of genetic testing is changing the way researchers define the presence of disease, however, and recent European guidelines have taken this into account, recognising milder, early phenotypes that do not meet conventional diagnostic criteria for DCM but are likely to be on the spectrum of genetic DCM [82]. Early genetic testing (currently through cascade screening) permits the identification of genotype-positive but phenotype-negative (‘G + P–’) individuals. This is most developed in HCM, an important parallel for future work in DCM. In one study of G + P– individuals with sarcomeric HCM mutations, this group of individuals manifested subtle, subclinical disease [83, 84], showing early markers of the disease and suggesting potential therapeutic targets.

## Conclusions

Advances in the genetics of DCM and systolic heart failure have highlighted numerous rare variants linked to DCM and fewer common variants linked to DCM and systolic heart failure. DCM and heart failure can be considered to lie at opposite ends of a spectrum—at one end DCM, where genetic contributions are most commonly due to single gene defects, and at the other end heart failure, a nebulous term encompassing a final common pathway resulting from a variety of individually small-effect-size genetic and environmental insults.

Within common variant discovery, the identification of systolic heart failure susceptibility variants expressed in the kidney or affecting inflammatory pathways reminds us of the complexity of the genetics of heart failure, and finding narrow therapeutic targets for such a global condition will be a key challenge.

Advances in rare variant discovery have been most notable for DCM, with the increasing identification of genes linked to DCM. These discoveries have the scope to provide novel insights into the pathogenesis of disease. However, as we broaden the number of genes to consider for heart failure syndromes, there will be a large increase in the number of variants of uncertain significance that are identified. Maintaining carefully curated disease databases such as ClinVar is a major undertaking, and it is unlikely that such curation can keep pace with the rate of sequencing. To help address some of these challenges, we can draw upon shared resources such as ExAC (gnomAD) to understand the background population-level variation, which has previously confounded the study of rare diseases. Familiarity with these resources will be essential in navigating the complex genetic architecture of both DCM and systolic heart failure in the future.

Genetic advances are informing new approaches for clinical management of patients with DCM and have highlighted the importance of considering genetic testing in all patients with DCM, not just those with a family history. Challenges remain in establishing clear genotype–phenotype correlations and in translating genetic advances into improvements in patient care for risk stratification or the development of novel therapies. In the short term, the field would benefit greatly from standardized phenotyping of both DCM and systolic heart failure using imaging and clinical criteria to ensure parity across studies.

## Box 1. Glossary

Arrhythmogenic right ventricular cardiomyopathy (ARVC)—a heart muscle condition leading to functional impairment of the right ventricle and arrhythmias.

Desmosome—intercellular junctions of cardiomyocytes.

Dilated cardiomyopathy (DCM)—a heart muscle condition leading to left ventricular dilation and systolic impairment.

Electrocardiogram (ECG)—a non-invasive surface recording of the electrical activity of the heart.

Ejection fraction (EF)—a numeric estimate of cardiac function based on the percentage of blood expelled from the right or left ventricle per heart beat. Cut-offs for left ventricular ejection fraction (LVEF) can be used to define heart failure syndromes. Normal LVEF is >55%.

Genome-wide association study (GWAS)—an unbiased approach, using regression analysis, to assess for the association between common polymorphisms and disease status/quantitative trait.

Heart failure—a clinical syndrome of symptoms and signs caused by impaired cardiac function. Predominantly left-sided systolic dysfunction, but can be right-sided systolic impairment and left-sided diastolic impairment.

Heart failure preserved ejection fraction (HFpEF)—heart failure caused by left ventricular diastolic impairment. Systolic function is preserved, with ejection fraction >50%. Previously termed diastolic heart failure.

Heart failure reduced ejection fraction (HFrEF)—heart failure caused by left ventricular systolic impairment. Previously termed systolic heart failure.

Hypertrophic cardiomyopathy (HCM)—a heart muscle condition leading to abnormal thickening (hypertrophy) of the left ventricle.

Left ventricular systolic dysfunction (LVSD)—impaired systolic function/reduced left ventricular ejection fraction. Can occur in the absence of symptoms. Does not imply one particular etiology.

Logarithm (base 10) of odds (LOD)—a statistical test of genetic linkage. A LOD score of >3 is conventionally considered evidence of linkage.

Sarcomere—the contractile unit of muscle, comprising thick and thin filaments.

Single-nucleotide polymorphism (SNP)—a variation in a single nucleotide in the genome, at a position where variation occurs in >1% of the population.

Titin gene (*TTN*)—gene coding for the largest human protein, expressed in cardiac and skeletal muscle; the leading genetic cause of DCM.

Z-disc—marks the lateral borders of the sarcomere, the point at which the thin filaments attach.

## Abbreviations

ARVC: Arrhythmogenic right ventricular cardiomyopathy
DCM: Dilated cardiomyopathy
ECG: Electrocardiogram
G + P–: Genotype-positive but phenotype-negative
GWAS: Genome-wide association study
HCM: Hypertrophic cardiomyopathy
HFpEF: Heart failure with preserved ejection fraction
HFrEF: Heart failure with reduced ejection fraction
HGMD: Human Gene Mutation Database
iPSC: Induced pluripotent stem cell
LD: Linkage disequilibrium
LVEF: Left ventricular ejection fraction
MAF: Minor allele frequency
PSI: Percentage spliced in
SNP: Single-nucleotide polymorphism
TTN: Titin gene
VUS: Variant of uncertain significance
